# Long non-coding RNA MALAT-1 modulates metastatic potential of tongue squamous cell carcinomas partially through the regulation of small proline rich proteins

**DOI:** 10.1186/s12885-016-2735-x

**Published:** 2016-09-01

**Authors:** Zhengyu Fang, Shanshan Zhang, Yufan Wang, Shiyue Shen, Feng Wang, Yinghua Hao, Yuxia Li, Bingyue Zhang, You Zhou, Hongyu Yang

**Affiliations:** 1 Biomedical Research Institute, Shenzhen Peking University- The Hong Kong University of Science and Technology Medical Center, Shenzhen, Guangdong province China; 2 Department of Oral and Maxillofacial, Shenzhen Hospital, Peking University, Shenzhen, Guangdong Province People’s Republic of China

**Keywords:** Tongue squamous cell cancer, Long non-coding RNA, MALAT-1, Cancer metastasis

## Abstract

**Background:**

We previously described several abnormally expressed long non-coding RNA (lncRNA) in tong squamous cell carcinomas (TSCCs) that might be associated with tumor progression. In the present study, we aimed to investigate the role of abnormally expressed metastasis-associated lung adenocarcinoma transcript 1 (MALAT-1) lncRNA in the metastatic potential of TSCC cells and its molecular mechanisms.

**Methods:**

Expression levels of MALAT-1 lncRNA were examined via quantitative reverse transcriptase polymerase chain reaction (qRT-PCR) in 127 TSCC samples as well as paired adjacent normal tissues and lymph node metastases (if exist). Lentiviral vectors expressing short hairpin RNA (shRNA) were used to knock down the expression of MALAT1 gene in two TSCC cell lines (CAL27 and SCC-25) with relatively higher MALAT-1 expression. Proliferational ability of the TSCC cells was analyzed using water soluble tetrazolium-1 (WST-1) assay. Metastatic abilities of TSCC cells were estimated in-vitro and in-vivo. We also performed a microarray-based screen to identify the genes influenced by MALAT-1 alteration, which were validated by real-time PCR analysis.

**Results:**

Expression of MALAT-1 lncRNA was enhanced in TSCCs, especially in those with lymph node metastasis (LNM). Knockdown (KD) of MALAT-1 lncRNA in TSCC cells led to impaired migration and proliferation ability in-vitro and fewer metastases in-vivo. DNA microarray analysis showed that several members of small proline rich proteins (SPRR) were up-regulated by KD of MALAT-1 lncRNA in TSCC cells. SPRR2A over-expression could impair distant metastasis of TSCC cells in-vivo.

**Conclusion:**

Enhanced expression of MALAT-1 is associated with the growth and metastatic potential of TSCCs. Knock down of MALAT-1 in TSCCs leads to the up-regulation of certain SPRR proteins, which influenced the distant metastasis of TSCC cells.

**Electronic supplementary material:**

The online version of this article (doi:10.1186/s12885-016-2735-x) contains supplementary material, which is available to authorized users.

## Background

Oral cancer is the third most common cancer in developing nations and the sixth most common cancer worldwide [1, 2]. Squamous cell carcinoma is the most common oral cancer and frequently involves the tongue [3–5]. Although tongue squamous cell carcinoma (TSCC) can be cured with proper treatment when detected early, patients who have had TSCC have a high risk of developing secondary and/or recurrent tumors in the surrounding area, a phenomenon called field effect. Once tumor cells spread to the lymph nodes, the overall mortality rate is high and the 5-year overall survival rate does not exceed 50 % [6–8].

Long non-coding RNAs (lncRNAs, pseudogenes and circRNAs) have recently come into light as powerful players in cancer pathogenesis and it is becoming increasingly clear that they have the potential of greatly contributing to the spread and success of personalized cancer medicine [9, 10]. In our previous study, we identified several lncRNAs that might be associated with the progression of TSCCs in a certain number of TSCC cases, which includes MALAT-1 [11]. MALAT-1 is a novel large, noncoding RNA. The MALAT-1 gene, also known as the NEAT2 gene, is found on chromosome 11q13 and is well- conserved among mammalian species [12]. The MALAT-1 transcript is widely expressed in normal human and mouse tissue, has been shown to localize to the nucleus and its 3′ end can be processed to yield a tRNA-like cytoplasmic RNA. MALAT-1 has been shown to be a potentially generic marker for epithelial carcinomas and is greatly up-regulated in lung adenocarcinoma metastasis [13], endometrial stromal sarcoma of the uterus [14], non-hepatic human carcinomas [15] and was recently reported to be overexpressed in placenta previa and to play a role in trophoblast invasion regulation [16].

In the present study, we enrolled additional TSCC patients and examined the expression levels of MALAT-1 in all the collected samples. We explored the correlation between the MALAT-1 lncRNA expression and cancer metastasis. We also aimed to find out the differentially expressed genes between MALAT-1 knockdown and control cells by DNA microarray analysis. We found that the expression of small proline-rich protein 2A (SPRR2A) were negatively regulated by MALAT-1 expression and had an influence on cancer metastasis in vivo.

## Methods

### Patients and tissue collection

This study was approved by Ethics Committee of Peking University Health Science Center (IRB00001053-08043). TSCC samples were obtained from 127 patients of the Department of Oral & Maxillofacial Surgery, Shenzhen Hospital, Peking University. A summary of cohort characteristics was listed in Table 1. A detailed description of tumor characteristics was listed in Additional file 1: Table S1. Adjacent normal mucosa tissues located at least 1.5 cm far from the macroscopically unaffected margins of the tumor were defined as normal controls. All the TSCC samples were graded in 4 groups according to common criteria of SCC staging: Stage1 (less than 2 centimeters in size and has not spread to lymph nodes in the area; *n* = 23), Stage2 (more than 2 cm in size, but less than 4 cm, and has not spread to lymph nodes in the area; *n* = 55), Stage3 (more than 4 cm in size/ has spread to only one lymph node on the same side of the neck as the cancer; *n* = 38), Stage4 (has spread to tissues around the lip and oral cavity/ has spread to more than one lymph node on the same side of the neck as the cancer, to lymph nodes on one or both sides of the neck, or to any lymph node that measures more than 6 cm/ has spread to other parts of the body, *n* = 11). The TSCC tissues were collected from patients undergoing surgical excision. Matched samples of TSCC (*n* = 127) and normal oral squamous cell mucosa (*n* = 127) were subjected to real-time PCR analysis. All patients were informed about the aims of specimen collection and gave signed written consent in accordance with the ethical guidelines of Peking University.

**Table 1:** Summary of the cohort characteristics

Characteristics	Information	
Gender	Female	46
	Male	81
Age	Average age	51.2
	Range	23 ~ 75
Tumor Location	Root	15
	Lateral margin	50
	Inferior surface	52
	Dorsum	3
	Around tongue tip	7
Lymph node metastasis (LNM)	With LNM	59
	Without LNM	68

### RNA extraction and real-time PCR

Total RNA was isolated from tissues by using a AxyPrepTM Blood Total RNA MiniPrep Kit (Axygen, US) according to the manufacturer’s instruction. First strand cDNA was synthesized with a RevertAidTM First Stand cDNA Synthesis Kit (Fermentas, US) using random hexamar primer. Quantitative PCR was performed through BioRad Chromo4 real-time PCR system. The primer sets for amplifying MALAT-1 and other related genes were listed in Table 2. Since “housekeeping” gene may have differential expression in the tissue types being evaluated [17], we compared the expression of 16 reference genes in 30 paired TSCC, ANT and LNM samples (Additional file 2: Figure S1). The sequences of the selected reference genes were listed in the Additional file 1: Table S2. We selected ACTB as the reference gene in analyzing the results. At the end point of PCR cycles, melt curves were made to check product purity. The level of MALAT-1 was expressed as a ratio relative to the β-actin mRNA in each sample. Exploratory data analysis using box plot was applied to visually identify the expression level of target mRNA.

**Table 2:** Primer sets used for amplifying the fragment of lncRNA transcripts and control

	Forward(5′–3′)	Reverse(5′–3′)
MALAT1	GGATCCTAGACCAGCATGCC	AAAGGTTACCATAAGTAAGTTCCAGAAAA
SPRR2A	GGATATTTGGCTCACCTCGT	GGAGAAAGAAGCTCCCTGTG
SPRR2D	CTGTAGTACACATCACTTGTGGC	ACTTGCATCCCAGGACAGAT
SPRR2E	CACAGCTTCACCTGCATCTT	CAATATGGCAGCCTCAGAAA
SPRR1B	GGCCACCAGATGCTGAAT	CAGAATGCTAATTGCAAGGC
ACTB	GAGCACAGAGCCTCGCCTTT	TCATCATCCATGGTGAGCTGGC

### Cell culture

Human tongue squamous cell carcinoma cell line CAL 27 and SCC-25 (CRL-2095™ & CRL-1628™) was obtained from the Cell Bank of the Chinese Academy of Sciences (Shanghai, China) where they were characterized by mycoplasma detection, DNA -Fingerprinting, isozyme detection and cell vitality detection. These cell lines were purchased in August 2012 and immediately expanded and frozen so that they could be restarted every 3 to 4 months from a frozen vial of the same batch of cells. CAL 27 and SCC-25 cells were cultured in Dulbecco’s modified Eagle’s medium (DMEM, GIBCO, US) supplemented with 10 % fetal bovine serum (PAA) and 1 % penicillin/ streptomycin (Life Technologies Inc., US).

### MALAT-1 knockdown by lentiviruses

To generate lentiviruses expressing MALAT-1 shRNA and control shRNAs, HEK293T cells grown on 10 cm dish were transfected with 6 μg of MALAT-1 shRNAs (cloned in PLKO.1) or control vector, 6 μg of pREV, 6 μg of pGag/Pol, and 2 μg of pVSVg. 12 h after transfection, cells were cultured with DMEM medium containing 20 % FBS for an additional 36 h. The culture medium containing lentivirus particles was centrifuged at 10000 × g for 2 min and then used for infection. 24 h after infection, cells were cultured with fresh medium for another 24 h, followed with further experiment. The knockdown efficiency was evaluated by real-time PCR analysis. The shRNA sequences targeting MALAT-1 are “ATG GAG GTA TGA CAT ATA AT” and “GGG AGT TAC TTG CCA ACT TG” [18].

### Cell proliferation assay

Cell proliferation was measured by Cell Proliferation Reagent WST-1 (Roche, USA) as introduced previously [19]. Cells were counted and plated in 96-well culture plates (1 × 10^3^ per well); WST-1 assay measuring the activity of mitochondrial dehydrogenases was performed following the manufacturer’s instructions at 0-, 1-, 2-, 3-, and 4-day time points.

### Cell migration assay

Migration assays were performed using 24-well Trans-well units with 8 mm pore size polycarbonate inserts (BD Biosciences, US). Trans-wells were coated overnight with 10 mg/ml of fibronectin in PBS at 48 °C, followed by incubation with 1 % BSA for 1 h at 37 °C. The SCC-25 and CAL27 cells transfected with shRNA (MALAT-1 shRNA) or plasmids (SPRR expression vectors and mock vectors) were detached with trypsin/EDTA, washed once with DMEM containing 10 % FBS, and re-suspended in DMEM containing 1 % FBS at 2 × 10^5^ cells/ml. Aliquots (100 microliters) of cell suspensions were directly added to the upper side of each chamber. Following incubation for 12 h, the cells on the upper side of the membrane were removed, whereas the cells that migrated to the underside were fixed with 3 % formaldehyde and stained with 0.3 % crystal violet for 10 min. The number of cells on the underside of the membrane was counted in five different fields with a light microscope at 100×, and the mean and SD was calculated from three independent experiments.

### DNA microarray

After washing the cells with 50 mM potassium phosphate buffer (pH 7.4), the total RNA of each sample was extracted by RNeasy Mini Kit (Qiagen, US). The procedure for the extraction of the total RNA was according to the manufacturer’s instruction. The quality of the extracted RNA was confirmed with Bioanalyzer 2100 (Agilent Technologies, US). GeneChip(R) arrays (Affymetrix) were used as the DNA microarrays. DNA microarray analysis was performed with Bio Matrix Research. Statistical analysis after data acquisition and normalization of expression data was performed using GeneSpring (Agilent Technologies, US). For the pathway- or function-based category classification, the Munich Information Center for Protein Sequence (MIPS) was used.

### Western blotting

Cells were washed with PBS and lysed in a buffer containing 50 mM Tris-HCl (pH 6.8), 2 % SDS, 10 % glycerol, phosphatase inhibitors (100 mM Na_3_VO_4_, 10 mM NaF) and protease inhibitor (1 mM PMSF). Equal amounts of protein were loaded on a SDS-PAGE and transferred to PVDF membrane. After blocking with 5 % non-fat milk in TBS-T (containing 0.1 % Tween-20), the membranes were incubated with specific primary antibodies, followed by HRP-conjugated secondary antibodies. Proteins were visualized by fluorography using an enhanced chemiluminescence system. Antibodies for SPRR1B, 2A (Abcam, US), 2E (Abnova, US) and β-actin (Sangon,Shanghai, China) were purchased as the primary antibodies for the approach.

### Establishment of the SCC metastases animal model in nude mice

The animal experiments were approved by the Ethics Committee of Peking University Health Science Center (IRB00001053-09028). Six-week-old male nude mice (Zi Guang Laboratory Animal Technology Co. Ltd., Guangdong, China) were placed under general anesthesia with 1 % pentobarbital sodium (Sigma). SCC-25/CAL 27 cells (5 × 10^6^) were injected subcutaneously (15 mice each group, and additional 15 mice for CAL27-Mock and CAL27-MALAT1KD cells). Metastasis was assayed by gross examination at autopsy and by PCR for Alu sequences in various organs. Control cells including SCC-25 and CAL27 cells caused grossly evident metastasis within the first 8 weeks and all animals were sacrificed at this time point. On the contrary, mice receiving MALAT-1 shRNA-transfectants were healthy at 8 weeks, but several were sacrificed for comparison, while the remaining mice were followed for an additional 4 weeks to determine if metastatic tumors developed. The volume of xenograft was calculated as v = 3/4πab^2^ (a = length, b = width). The average volume of the xenografts at sacrifice were listed in the Additional file 1: Table S3. Grossly obvious tumors and metastases were dissected and fixed immediately with 4 % paraformaldehyde for pathological analysis (Some of the animal models as well as metastases were shown in the Additional file 3: Figure S2).

### Plasmids and transfection

The cloned SPRR1B & 2A cDNA fragment were inserted into pcDNA3.1 expression vector to construct the expression vectors. To produce stable transfectants, pcDNA-SPRR1B & 2A as well as mock plasmids were stably transfected into the CAL27/SCC25 line using Lipofectamine 2000 reagent (LF2000, Invitrogen, Carlsbad, CA) according to the manufacturer’s recommendations. Selection was performed via the addition of 1 mg/ml G418. The transfectants from the backbone vector and pcDNA3-SPRR1B/2A were designated as mock-CAL27/SCC25 and SPRR1B/2A-CAL27/SCC25, respectively.

### Statistical analysis

GraphPad Prism software (Version 5.0) was used to analyze the obtained data. Results of the MALAT-1 lncRNA expression for paired TSCC and ANT samples or paired TSCC and local lymph-node metastasis were compared using paired *t*-test. Results of the MALAT-1 lncRNA expression for different TSCC groups were compared using non-parametric Mann-Whitney test. Data of in-vitro experiments were analyzed using the chi-square test or Fisher exact test. Differences of the metastasis between different groups of mouse models were analyzed using Chi-square test. *P*-values less than 0.05 were considered statistically significant.

## Results

### Enhanced expression of MALAT-1 lncRNA correlates with lymph node metastasis in TSCCs

**Fig. 1:**
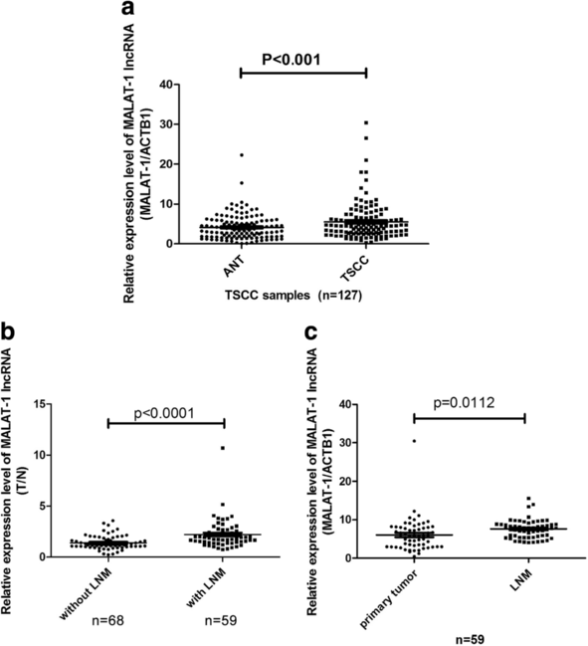
Enhanced expression of MALAT-1 lncRNA in TSCC. Real-time PCR assay was carried out as described under Methods Section and the results were obtained from indicated group of samples. **a** Scatter plot illustrated the relative expression of MALAT-1 as a ratio of lncRNA to β-actin mRNA in each sample; **b** Scatter plot illustrated the relative expression of MALAT-1 as a ratio of TSCC to paired ANT in the TSCCs with or without lymph node metastasis; **c** Scatter plot illustrated the relative expression of MALAT-1 as a ratio of lncRNA to β-actin mRNA in each sample

As a complementary experiment for the previous study, we examined the expression of MALAT-1 lncRNA in all the collected TSCC samples (*n* = 127), paired adjacent normal tissues (ANTs) and lymph node metastases (*n* = 59) in the present study. As shown in Fig. 1a, the expression levels of MALAT-1 lncRNA increased significantly in TSCCs compared to paired ANTs. In TSCC tissues with lymph node metastasis (LNM), the expression levels of MALAT-1 lncRNA were statistically higher than those without LNM (Fig. 1b). On the other hand, the differences were less significant between paired primary tumor and LNMs (*n* = 59, Fig. 1c).

### Knockdown of MALAT-1 lncRNA impaired migration of TSCC cells in-vitro and in-vivo

**Fig. 2:**
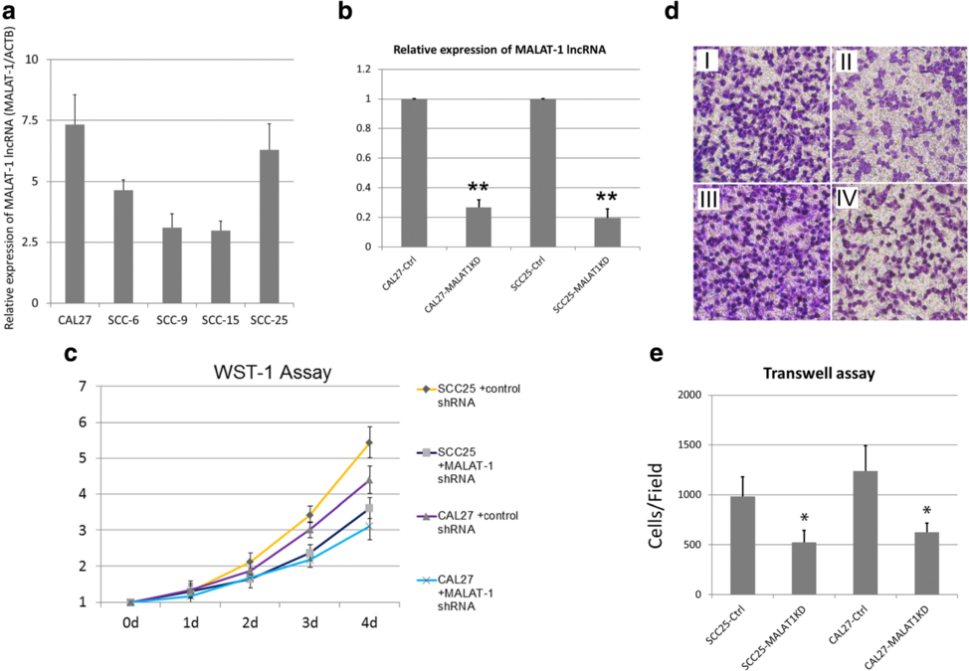
Knockdown of MALAT-1 lncRNA impaired proliferation and migration of TSCC cells in-vitro. **a** Expression levels of Malat-1 lncRNA were examined by real-time PCR. **b** After treatment of lentiviruses expressing MALAT-1 shRNA and control shRNAs, the expression levels of MALAT-1 lncRNA were examined by real-time PCR. The relative expression of Malat-1 lncRNA (as the ratio of Malat-1 lncRNA to β-actin mRNA) is illustrated as a ratio to control (cells transfected with nonsense siRNA). **c** WST-1 (Roche) assay measuring the activity of mitochondrial dehydrogenases was performed following the manufacturer’s instruction at 0-, 1-, 2-, 3-, 4- day time points. Error bars represent the standard deviation of the mean; **d** Cell migration was determined using a transwell assay as described in the Methods section. Microscopic image of migrated CAL 27 and SCC-25 cells with indicated treatments: (I) SCC25 + control shRNA; (II) SCC25 + MALAT1KD shRNA; (III) CAL27 + control shRNA; (IV) CA L27 + MALAT1KD shRNA; **e** Diagrams of migrating cells from the different transfectants are shown, which are from more than three independent experiments.*P < 0.05 versus control

In the preliminary work, we found that the expression levels of MALAT-1 were higher in SCC25 and CAL27 lines than those in SCC-6, SCC-9 and SCC15 lines (Fig. 2a). Thus, we selected these two cells for the in-vitro studies. After MALAT-1 was knock down by lentiviruses (Fig. 2b), the cell growth were both attenuated in SCC25 and CAL27 cells (Fig. 2c). We next estimated cell migration of SCC25 and CAL27 cells using trans-well assay. It was found that the both SCC25 and CAL27 cells with impaired expression of Malat-1 migrated less effectively through trans-well membrane (Fig. 2d & e).

We next tested the metastatic potential of control shRNA and MALAT-1 shRNA transfectants 8–12 weeks after subcutaneous injection as introduced in the Methods section. Decreased number of mice that developed metastasis was observed in CAL27-MALAT1KD group compared to the control group (Table 3, *p* < 0.05). Detailed information of organ-specific metastases was also listed in Table 3. On the other hand, the results using SCC-25 cells could hardly be analyzed due to the insufficient metastasis formation. Thus, we selected CAL27 cells for following the in-vivo experiments.

**Table 3:** The number of organ-specific metastasis sites in nude mice after cell plantation

Metastasis site	CAL-27-Mock (30mice/group)	CAL27-MALAT1KD (30 mice/group)	SCC-25-Mock (15mice/group)	SCC-25-MALAT1KD (15 mice/group)
Brain	0 (0 %)	0 (0 %)	0 (0 %)	0 (0 %)
Kidney	5 (16.7 %)	2 (6.7 %)	1 (6.7 %)	0 (0 %)
Liver	9 (30 %)	4 (13.3 %)	2 (13.3 %)	2 (13.3 %)
Mediastinum	4 (13.3 %)	1 (3.3 %)	0 (0 %)	0 (0 %)
Bone	3 (10 %)	0 (0 %)	0 (0 %)	0 (0 %)
Colon	14 (46.7 %)	6 (20 %)*	4 (0 %)	2 (0 %)
Local invasion	22 (73.3 %)	18 (60 %)	12 (0 %)	13 (0 %)
Mesentery	7 (23.3 %)	3 (10 %)	3 (13.3 %)	1 (6.7 %)
Mice with metastases	18 (60 %)	9 (30 %)*	5 (33.3 %)	2 (13.3)

*P < 0.05 V.S. CAL27-Mock group

### Knockdown of MALAT-1leads to the enhanced expression of several SPRR proteins

**Fig. 3:**
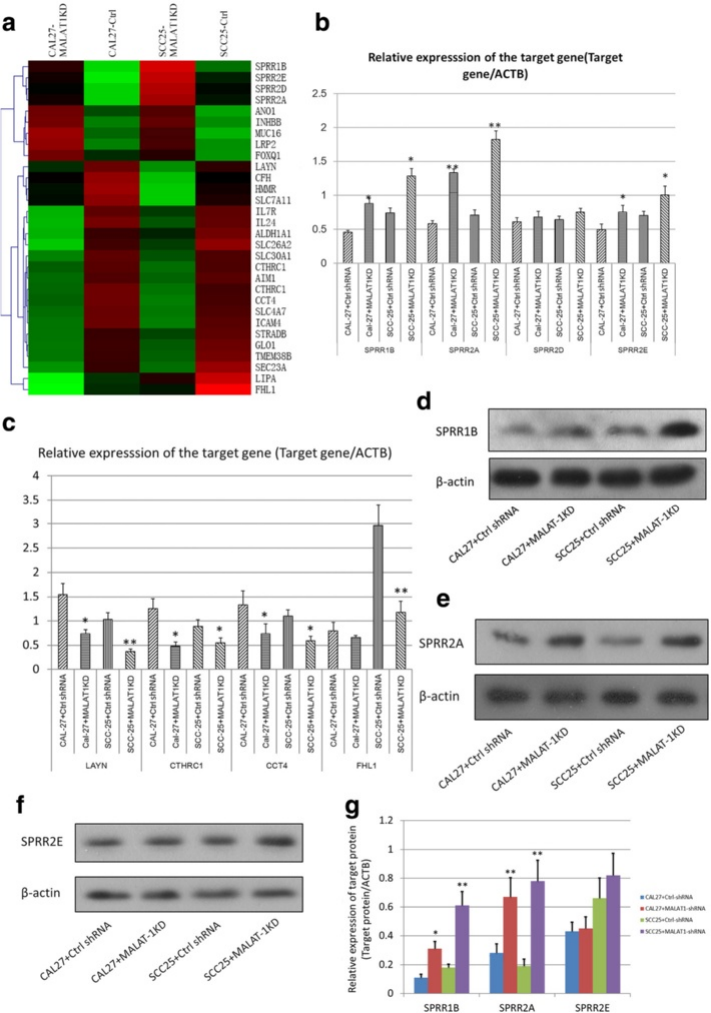
Knockdown of MALAT-1 leads to the enhanced expression of SPRR proteins. **a** The heatmap illustrated the genes most significantly influenced by KD of MALAT-1 using microarray analysis. **b** & **c** Real-time PCR analysis was carried out to examine the mRNA expression of selected genes screened by microarray analysis;*P < 0.05 versus control; **P < 0.01 versus control. **d**, **e** & **f** Western blotting was performed to examine the protein levels of SPRR1B, 2A &2E in CAL 27 and SCC-25 cells; β-actin was used as control. **g** The histogram shows the mean ± SD of the gray scale analysis, which were obtained from 3 independent experiments each group; *P < 0.05;**P < 0.01

As a non-coding RNA, MALAT-1 could not directly influence cell migrational ability. We surveyed the differentially expressed genes between MALAT-1 KD and control cells by DNA microarray analysis. Numerous genes showing significant differential expression were identified in the microarray analysis in two independent MALAT-1 KD cell lines. The down-regulated genes in MALAT-1 KD cells included genes previously implicated in extracellular matrix and cytoskeleton regulation, such as *LAYN, CCT4, CTHRC1,* and *FHL1.* Here we noticed that expressions levels of several members of SPRR family were also influenced by MALAT-1 KD (Fig. 3a), which was a novel finding.

The qRT-PCR analysis was performed to confirm the expression level of differential expressed genes. As shown in Fig. 3b, mRNA levels of SPRR1B, SPRR2A, and SPRR2E were significantly up-regulated in MALAT-1 KD cells. The altered expression of *LAYN, CCT4, CTHRC1,* and *FHL1* were also confirmed by qRT-PCR (Fig. 3c). We also used a Western blot to examine the protein levels of these genes. It was found that the protein levels of SPRR1B and 2A were significantly induced in MALAT-1 KD cells (Fig. 3d, e & g), while SPRR2E were slightly influenced (Fig. 3f & g).

### Over-expression of SPRR2A prevents TSCC metastasis in-vivo

**Fig. 4:**
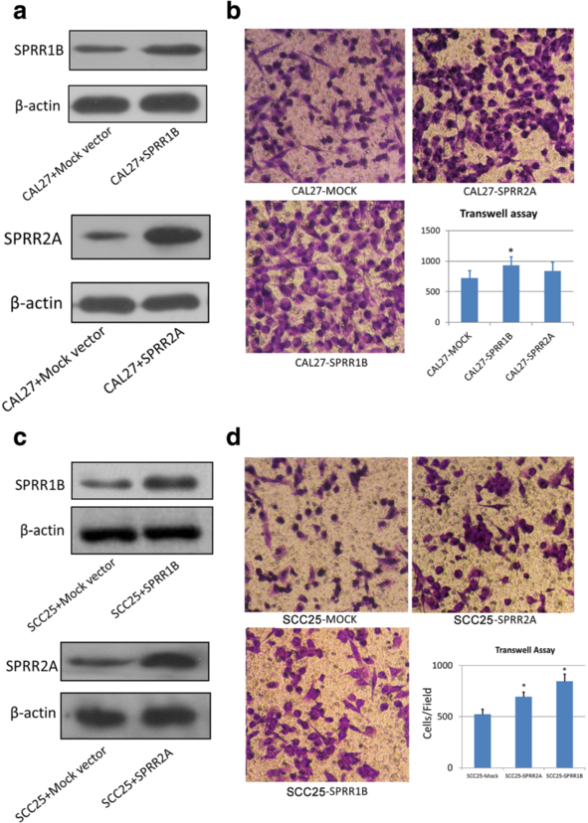
SPRR2A promotes TSCC migration in-vitro. **a** & **c** Western blotting was performed to examine the protein levels of SPRR1B & 2A in the targeted cells; β-actin was used as control. **b** & **d** Cell migration was determined using a transwell assay as described Fig. 2c (the incubation time of the cells here was adjusted to 8 h to avoid high density). Diagrams of migrating cells from the different are shown, which are from more than three independent experiments.*P < 0.05 versus control

Previously, it was indicated that *LAYN, CCT4, CTHRC1,* and *FHL1* gene were correlated with the migrational potential of lung cancer cells [13]. Here we wondered whether SPRRs regulated by MALAT-1 also could influence TSCC metastasis. SPRRs are a subclass of structural proteins which constitute cornified cell envelope precursors. Several studies have suggested that the SPRRs are related to increased epithelial proliferation and malignant processes. Here we first use trans-well assay to estimate the migrational/invasive abilities of TSCC cells with different expression of SPRR1B and 2A. As shown in Fig. 4a & c, SPRR2A/1B transfectants showed marked increase of protein levels in CAL27 and SCC25 cells. In-vitro studies showed that over-expression of SPRR1B and 2A slightly promoted the migration of CAL 27 cells and SCC25 cells (Fig. 4b & d) and had little effects on cell proliferation (Additional file 4: Figure S3). We next tested the metastatic potential of mock vector and SPRR2A/1B transfectants 8–12 weeks after subcutaneous injection. SPRR2A-CAL27 cells showed impaired distant metastasis compared to Mock-CAL27 cells (Table 4), while no obvious differences were observed between SPRR1B-CAL27 cell and mock cells. Thus, increased MALAT-1 expression might enhance TSCC distant metastasis partially through the down-regulation of SPRR2A.

**Table 4:** The number of organ-specific metastasis sites in nude mice after cell plantation (15 mice/each group)

Metastasis site	Mock-CAL27	SPRR2A-CAL27	SPRR1B-CAL27
Brain	0	0	0
Kidney	1	0	2
Liver	6	2	7
Mediastinum	2	1	2
Bone	2	0	2
Colon	8	3	9
Local invasion	11	13	12
Mesentery	3	1	5
Mice with metastases	11 (73.3 %)	5 (33.3 %)*	12 (80 %)

*P < 0.05 V.S. Mock-CAL27 group

## Discussion and conclusions

LncRNA contributes significantly to human transcriptome and is believed to play a critical role in cancer development. A previous report showed that ~60 % of the detected lncRNAs have aberrant expressions in oral premalignant lesions [20]. Previously we focused on TSCC and a series of abnormally expressed cancer-related lncRNAs were identified [11]. Here we further proved that the expression levels of MALAT-1 lncRNA were markedly elevated in TSCC, especially in TSCC with LNM. In TSCCs with LNM, increased expression of MALAT-1 lncRNA was detected in LNMs than in primary tumors. Cell growth and migration was attenuated in MALAT1-KD TSCC cells. These all indicated the potential role of MALAT-1 lncRNA in metastasis of TSCCs.

In microarray analysis, we found that MALAT-1 knockdown led to the accumulation of SPRR proteins, which was a novel finding. The SPRRs constitute cornified cell envelope precursors [21]. Several studies have suggested that the SPRRs are related to increased epithelial proliferation and malignant progression [22]. Why knockdown of MALAT-1 lncRNA would lead to the accumulation of SPRR proteins in TSCC cells? One possibility is that MALAT1 regulates gene transcription via modification of the epigenetic program. Yang et al. reports MALAT1 can facilitate the assembly of multiple co-repressors/co-activators and finds that MALAT1 alters the histone modifications on chromatin by alternating the activity of Polycomb2 protein (Pc2) [23]. In addition, MALAT1 molecule has been linked to the physical interaction with critical chromatin-modifier Polycomb Repressive Complex 2 (PRC2) to modulate the epigenetic status of target genes [24]. Hirata H. et al. [25] reports that MALAT1 directly binds to the EZH2 protein, which is a critical component of the PRC2 complex to play the methyltransferase activity of the chromatin histone modifications; similar result showed that MALAT1 binds to active chromatin sites [26]. These experimental evidences showed that MALAT1 modulates the chromatin histone methylations by binding to PRC2 complex and abolishing its methylation activity.

Another possibility goes to the direct regulation of target gene by lncRNA. Four different regulation mechanisms by lncRNAs might be involved in MALAT1-mediated modulation: (a) MALAT-1 lncRNA molecule interacts with double strand DNA and represses gene transcription; (b) MALAT-1 lncRNA fragments act as intronic siRNA to bind with mRNA and repressing mRNA translation; (c) Produce alternative splicing lncRNAs to regulate gene expression. Different isoforms from alternative splicing have different regulation activity and specificity, which regulate the gene expression with different patterns; (d) MALAT-1 lncRNA molecule interacts with basal transcriptional machinery which disrupts the transcription initiation complex and represses transcription [27–29]. These need further investigation.

In the present study, over-expression of SPRR2A in TSCC cells could slightly promote cell migration in-vitro but impair distant metastasis in-vivo, which seemed to be a confusing result. A previous finding also showed that SPRR2A over-expression increases local tumor invasiveness but prevents metastasis in cholangiocarcinoma [30]. This may be explained by the irreversible epithelial-mesenchymal transition (EMT) of the SPRR2A transfectants. Progression of epithelial tumors requires temporary acquisition of mesenchymal characteristics (EMT), which allows for local invasion and hematogenous dissemination of the cancer cells. At distant sites, these cells undergo mesenchymal-epithelial transition (MET) to establish residence and form tumors that are histopathologically similar to the primary tumor. Dr. Specht et al. reported that their stable SPRR2A clones are in a permanent, irreversible mesenchymal state. In the current study, CAL27-SPRR2A cells also appeared to be plastic and have high mobility, which showed mesenchymal behavior (indicated by increased Twist protein expression in SPRR2A-CAL27 but not SPRR1B-CAL27, Additional file 5: Figure S4). Thus, impaired MET ability of SPRR2A-CAL27 might be associated with the reduced distant metastases.

In general, plausibly, our findings indicated that the expression level of MALAT-1 have the potential to indicate MALAT-1 have potential for prognostic indicator in lymph node metastasis of TSCC. MALAT-1 knockdown led to the accumulation of SPRR proteins, in which SPRR2A was shown to be associated with the distant metastasis of TSCCs. The underlying mechanisms of the regulation of SPRRs by MALAT-1 need to be extensively investigated in the future.

## Additional files

Additional file 1: Table S1. Detailed information of tumoral characteristics of patients and the information of metastasis. *The information of lymph node metastasis includes the metastatic site, number of lymph nodes involved and largest diameter of metastasis. **Table S2.** Primer sequences of the 16 reference genes. **Table S3.** Volume of the xenografts when the mice were sacrificed: The in-vivo experiments using mouse model were performed as introduced in the Methods section. The average values express as mean ± s.d. (DOCX 30 kb) Additional file 2: Figure S1. References gene selection for the paired TSCC, ANT and LNMs. A: Melting curve of the amplification of the targeted genes; B: Gel electrophoresis of the amplified products in **Figure S1A.**; C: Column diagram with SD bar illustrated the relative expression of targeted genes as a ratio of ANT/LNM to paired primary tumor. (JPG 718 kb) Additional file 3: Figure S2. Establishment of the SCC metastases animal model in nude mice; grossly obvious tumors and metastases were dissected and fixed immediately with 4 % paraformaldehyde for pathological analysis. (JPG 1505 kb) Additional file 4: Figure S3. WST-1 (Roche) assay measuring the activity of mitochondrial dehydrogenases was performed following the manufacturer’s instruction at 0-, 1-, 2-, 3-, 4- day time points. Error bars represent the standard deviation of the mean. (JPG 261 kb) Additional file 5: Figure S4. Western blotting was performed to examine the protein levels of Twist in the indicated cells; β-actin was used as control. (JPG 151 kb)

## Abbreviations

MALAT-1: Metastasis-associated lung adenocarcinoma transcript 1
TSCC: Tong squamous cell carcinoma
lncRNA: Long non-coding RNA
qRT-PCR: Quantitative reverse transcriptase polymerase chain reaction
shRNA: Short hairpin RNA
WST-1: Water soluble tetrazolium-1
LNM: Lymph node metastasis
SPRR: Small proline rich
KD: Knockdown
MIPS: Munich Information Center for Protein Sequence
ANT: Adjacent normal tissue
LAYN: Layilin
CCT4: Chaperonin containing TCP1 subunit 4
CTHRC1: Collagen triple helix repeat containing 1
FHL1: Four and a half LIM domains 1
