# Supplementary material for: Long non-coding RNA MALAT-1 modulates metastatic potential of tongue squamous cell carcinomas partially through the regulation of small proline rich proteins
Source: BMC Cancer. 2016 Sep 1;16(1):706. doi: 10.1186/s12885-016-2735-x (PMC5009554; doi:10.1186/s12885-016-2735-x)
Supplement: Additional file 1: Table S1. — Detailed information of tumoral characteristics of patients and the information of metastasis. *The information of lymph node metastasis includes the metastatic site, number of lymph nodes involved and largest diameter of metastasis. Table S2. Primer sequences of the 16 reference genes. Table S3. Volume of the xenografts when the mice were sacrificed: The in-vivo experiments using mouse model were performed as introduced in the Methods section. The average values express as mean ± s.d. (DOCX 30 kb) [file 12885_2016_2735_MOESM1_ESM.docx]

**Supplementary Table S1: Detailed information of tumoral characteristics of patients and the information of metastasis**

| **No.** | **Location** | **TNM** | **Lymph node metastasis*** | **Distant metastasis** |
| --- | --- | --- | --- | --- |
| 1 | lateral margin | T2N0M0 | none | none |
| 2 | around tongue tip | T2N0MO | none | none |
| 3 | lateral margin | T2N2MO | homolateral, single,4cm | none |
| 4 | root | T2N0MO |  | none |
| 5 | root | T3N0MO | none | none |
| 6 | lateral margin | T3N0MO | none | none |
| 7 | lateral margin | T2N1M0 | homolateral, single,2cm | none |
| 8 | dorsum | T2N0M0 | none | none |
| 9 | lateral margin | T3N1M0 | homolateral, single,2cm | none |
| 10 | root | T4N0M0 | none | none |
| 11 | dorsum | T2N0M0 | none | none |
| 12 | lateral margin | T2N0M0 | none | none |
| 13 | lateral margin | T2N0M0 | none | none |
| 14 | inferior surface | T3N2M0 | homolateral, multiple, ＜6cm | none |
| 15 | lateral margin | T2N1MO | homolateral single,2cm | none |
| 16 | lateral margin | T2N0M0 | none | none |
| 17 | lateral margin | T3N2M0 | homolateral, multiple,＜6cm | none |
| 18 | lateral margin | T3N2M0 | homolateral, single,5cm | none |
| 19 | lateral margin | T2N0MO | none | none |
| 20 | lateral margin | T2N0MO | none | none |
| 21 | root | T2N0MO | none | none |
| 22 | lateral margin | T3N2MO | homolateral, multiple, ＜6cm | none |
| 23 | lateral margin | T2N0M0 | none | none |
| 24 | dorsum | T2N0M0 | none | none |
| 25 | inferior surface | T3N0MO | none | none |
| 26 | lateral margin | T3N2M0 | homolateral, multiple, ＜6cm | none |
| 27 | inferior surface | T2N0M0 | none | none |
| 28 | root | T2N0M0 | none | none |
| 29 | lateral margin | T3N2MO | homolateral, multiple, ＜6cm | none |
| 30 | lateral margin | T3N2MO | homolateral, multiple, ＜6cm | none |
| 31 | lateral margin | T2N0M0 | none | none |
| 32 | root | T3N0MO | none | none |
| 33 | inferior surface | T2N0MO | none | none |
| 34 | inferior surface | T2N2M0 | homolateral, multiple, ＜6cm | none |
| 35 | lateral margin | T2N2M0 | homolateral, multiple, ＜6cm | none |
| 36 | inferior surface | T2N2M0 | homolateral, multiple, ＜6cm | none |
| 37 | around tongue tip | T2N0M0 | none | none |
| 38 | inferior surface | T2N1MO | homolateral, single,2cm | none |
| 39 | lateral margin | T2N0M0 | none | none |
| 40 | root | T2N0MO | none | none |
| 41 | inferior surface | T2N0M0 | none | none |
| 42 | lateral margin | T2N0M0 | none | none |
| 43 | lateral margin | T2N2M0 | homolateral, multiple, ＜6cm | none |
| 44 | lateral margin | T2N0M0 | none | none |
| 45 | lateral margin | T2N1MO | homolateral, single,2cm | none |
| 46 | root | T2N0M0 | none | none |
| 47 | lateral margin | T2N0M0 | none | none |
| 48 | lateral margin | T2N2MO | homolateral, multiple,＜6cm | none |
| 49 | lateral margin | T1N0M0 | none | none |
| 50 | lateral margin | T2N0MO | none | none |
| 51 | lateral margin | T2N0M0 | none | none |
| 52 | inferior surface | T3N2MO | homolateral, multiple,＜6cm | none |
| 53 | root | T3N2MO | homolateral, multiple,＜6cm | none |
| 54 | root | T2N0MO | none | none |
| 55 | inferior surface | T2N0MO | none | none |
| 56 | inferior surface | T3N2MO | bilateral, multiple, ＜6cm | none |
| 57 | inferior surface | T2N0MO | none | none |
| 58 | inferior surface | T2N0MO | none | none |
| 59 | inferior surface | T2N0MO | none | none |
| 60 | inferior surface | T2N0MO | none | none |
| 61 | inferior surface | T1N0M0 | none | none |
| 62 | inferior surface | T3N2MO | homolateral, multiple,＜6cm | none |
| 63 | inferior surface | T2N1MO | homolateral, single, 2cm | none |
| 64 | inferior surface | T2N0MO | homolateral, multiple,＜6cm | none |
| 65 | inferior surface | T2N0M0 | none | none |
| 66 | inferior surface | T2N2MO | homolateral, multiple,＜6cm | none |
| 67 | inferior surface | T2N2M1 | bilateral, multiple，<6cm | lung |
| 68 | inferior surface | T2N2MO | homolateral, multiple,＜6cm | none |
| 69 | inferior surface | T3N2MO | homolateral, multiple,＜6cm | none |
| 70 | lateral margin | T2N2MO | homolateral, multiple,＜6cm | none |
| 71 | inferior surface | T2N1MO | homolateral, single,3cm | none |
| 72 | inferior surface | T2N1MO | homolateral, single,2cm | none |
| 73 | around tongue tip | T3N1M0 | homolateral, single,3cm | none |
| 74 | inferior surface | T2N0MO | none | none |
| 75 | inferior surface | T1N0M0 | none | none |
| 76 | inferior surface | T2N1MO | homolateral, single,1cm | none |
| 77 | root | T2N0MO | none | none |
| 78 | lateral margin | T2N1MO | homolateral, single,2cm | none |
| 79 | lateral margin | T3N2M1 | bilateral, multiple <6cm， | lung |
| 80 | around tongue tip | T3N2M0 | homolateral multiple,＜6cm | none |
| 81 | inferior surface | T3N2M1 | bilateral, <6cm， | lung |
| 82 | inferior surface | T2N1MO | homolateral single,2cm | none |
| 83 | lateral margin | T2N1MO | homolateral single,1cm | none |
| 84 | inferior surface | T2N2M1 | bilateral, multiple, <6cm， | bone |
| 85 | inferior surface | T2N0M0 | none | none |
| 86 | inferior surface | T2N0M0 | none | none |
| 87 | lateral margin | T1N0M0 | none |  |
| 88 | lateral margin | T3N2M1 | bilateral, multiple, <6cm | bone |
| 89 | inferior surface | T2N0M0 | none | none |
| 90 | inferior surface | T2N2MO | homolateral, multiple,＜6cm | none |
| 91 | inferior surface | T3N2MO | homolateral, multiple,＜6cm | none |
| 92 | lateral margin | T2N2M0 | homolateral, multiple,＜6cm | none |
| 93 | root | T2N2M1 | bilateral, multiple, <6cm | liver |
| 94 | lateral margin | T2N1MO | homolateral, single,1cm | none |
| 95 | inferior surface | T2N2M0 | homolateral, multiple, ＜6cm | none |
| 96 | lateral margin | T2N2M0 | homolateral, multiple, ＜6cm | none |
| 97 | inferior surface | T2N2M0 | homolateral, multiple, ＜6cm | none |
| 98 | around tongue tip | T2N0M0 | none | none |
| 99 | inferior surface | T2N1MO | homolateral, single,2cm | none |
| 100 | lateral margin | T2N1M0 | homolateral, single,<6cm | none |
| 101 | root | T2N0MO | none | none |
| 102 | inferior surface | T2N0M0 | none | none |
| 103 | lateral margin | T2N0M0 | none | none |
| 104 | lateral margin | T2N2M1 | homolateral, multiple, ＜6cm | Bone |
| 105 | lateral margin | T2N0M0 | none | none |
| 106 | lateral margin | T2N1MO | homolateral, single,3cm | none |
| 107 | inferior surface | T2N0M0 | none | none |
| 108 | lateral margin | T2N0M0 | none | none |
| 109 | inferior surface | T2N2MO | homolateral, multiple,＜6cm | none |
| 110 | around tongue tip | T1N0M0 | none | none |
| 111 | inferior surface | T2N0MO | none | none |
| 112 | lateral margin | T2N0M0 | none | none |
| 113 | root | T3N2M1 | homolateral, multiple,＜6cm | Lung |
| 114 | inferior surface | T3N2MO | homolateral, multiple,＜6cm | none |
| 115 | lateral margin | T2N0MO | none | none |
| 116 | lateral margin | T2N0MO | none | none |
| 117 | inferior surface | T3N2MO | bilateral, multiple, ＜6cm | none |
| 118 | inferior surface | T2N0MO | none | none |
| 119 | around tongue tip | T2N0MO | none | none |
| 120 | inferior surface | T2N0MO | none | none |
| 121 | inferior surface | T2N0MO | none | none |
| 122 | inferior surface | T1N0M0 | none | none |
| 123 | root | T3N2MO | homolateral, multiple,＜6cm | none |
| 124 | lateral margin | T2N1MO | homolateral, single, 2.5cm | none |
| 125 | lateral margin | T2N0MO | homolateral, multiple,＜6cm | none |
| 126 | inferior surface | T2N0M0 | none | none |
| 127 | inferior surface | T2N2MO | homolateral, multiple,＜6cm | none |

*The information of lymph node metastasis includes the metastatic site, number of lymph nodes involved and largest diameter of metastasis.

**Supplementary Table S2: Table 1 Primer sequences of the reference genes.**

| **Gene Symbol** | **Official Full Name** | **NCBI Reference Sequence** | **Primer Sequence (5’ to 3’)** |
| --- | --- | --- | --- |
| **TBP** | TATA-binding protein | NM_003194 | F:GAATATAATCCCAAGCGGTTTG  R:ACTTCACATCACAGCTCCCC |
| **UBQLN1** | ubiquilin 1 | NM_013438 | F:ATGAGACAACAGCTCCCAACT  R:GGGCTTCCGTTGCTAATGTC |
| **EEF1A1** | elongation factor-1 alpha | NM_001402 | F:TATCCACCTTTGGGTCGCTT  R:GTGGGGTGGCAGGTATTAGG |
| **B2M** | beta-2-microglobulin | NM_004048 | F:AGATGAGTATGCCTGCCGTG  R:TCATCCAATCCAAATGCGGC |
| **KALPHA1** | tubulin, alpha 1b | NM_006082 | F:GTGCGTTACTTACCTCGACTCT  R:CACGCATAGTGGCTAGGGAT |
| **YWHAZ** | tyrosine 3-monooxygenase/tryptophan 5-monooxygenase activation protein, zeta polypeptide | NM_003406 | F:ACTTGACATTGTGGACATCGGA  R:AGGCCGGTTAATTTTCCCCT |
| **UBC** | ubiquitin C | NM_021009 | F:GGCGAGGAAAAGTAGTCCCT  R:AGAACTGCGACCCAAATCCC |
| **SDHA** | succinate dehydrogenase complex, subunit A,flavoprotein(Fp) | NM_004168 | F:TGCCATCCACTACATGACGG  R:GCTCTGTCCACCAAATGCAC |
| **GUSB** | beta-glucuronidase | NM_000181 | F:CTATGCCATCGTGTGGGTGA  R:TGGACCAGGTTGCTGATGTC |
| **GAPD** | glyceraldehyde 3-phosphate dehydrogenase | NM_002046 | F:TCCAAAATCAAGTGGGGCGA  R:TGATGACCCTTTTGGCTCCC |
| **PPIA** | peptidyl prolyl isomerase A | NM_021130 | F:GGTATAAAAGGGGCGGGAGG  R:CTGCAAACAGCTCAAAGGAGAC |
| **TIPRL** | TOR signaling pathway regulator | NM_152902 | F:TTACCATGAGGCTGACAAGACC  R:TGTGAGTCTGCTGGGTTAGGA |
| **POLR2A** | polymerase (RNA) II (DNA directed) polypeptide A | NM_000937 | F:GCTTCAGCCCAGGTTACTCC  R:AGGGACTCTGGGGTGTGTAG |
| **TUBA3** | tubulin, alpha 1a | NM_006009 | F:AAGCAGCAACCATGCGTGA  R:CTCCTCCCCCAATGGTCTTG |
| **HPRT1** | hypoxanthine phosphoribosyltransferase 1 | NM_000194 | F:CCCTGGCGTCGTGATTAGTG  R:TCGAGCAAGACGTTCAGTCC |
| **ACTB** | beta-actin | NM_001101 | F:GAGCACAGAGCCTCGCCTTT  R:TCATCATCCATGGTGAGCTGGC |

**Supplementary Table S3: Volume of the xenografts when the mice were sacrificed.**

| **Xenograft** | | **Sacrificed at 8month (mm^3^, number)** | **Sacrificed at 12 month (mm^3^, number)** |
| --- | --- | --- | --- |
| **Part 1 of the in-vivo experiments** | | | |
| **CAL27-Mock** | 1628.4±548.6 (n=30) | | N/A (n=0) |
| **CAL27-MALAT1KD** | 692.0±329.8 (n=11) | | 1457.9± 718.6 (n=19) |
| **SCC25-Mock** | 2168.4± 783.2 (n=15) | | N/A (n=0) |
| **SCC25-MALAT1KD** | 893.3±297.5 (n=5) | | 1948.9± 839.3 (n=10) |
| **Part 2 of the in-vivo experiments** | | | |
| **Mock-CAL27** | | 1733.5 (n=15) | N/A (n=0) |
| **SPRR2A-CAL27** | | 1596.9 (n=15) | N/A (n=0) |
| **SPRR1B-CAL27** | | 1926.0 (n=15) | N/A (n=0) |
