# Supplementary figures and images for: Long non-coding RNA MALAT-1 modulates metastatic potential of tongue squamous cell carcinomas partially through the regulation of small proline rich proteins
Source: BMC Cancer. 2016 Sep 1;16(1):706. doi: 10.1186/s12885-016-2735-x (PMC5009554; doi:10.1186/s12885-016-2735-x)

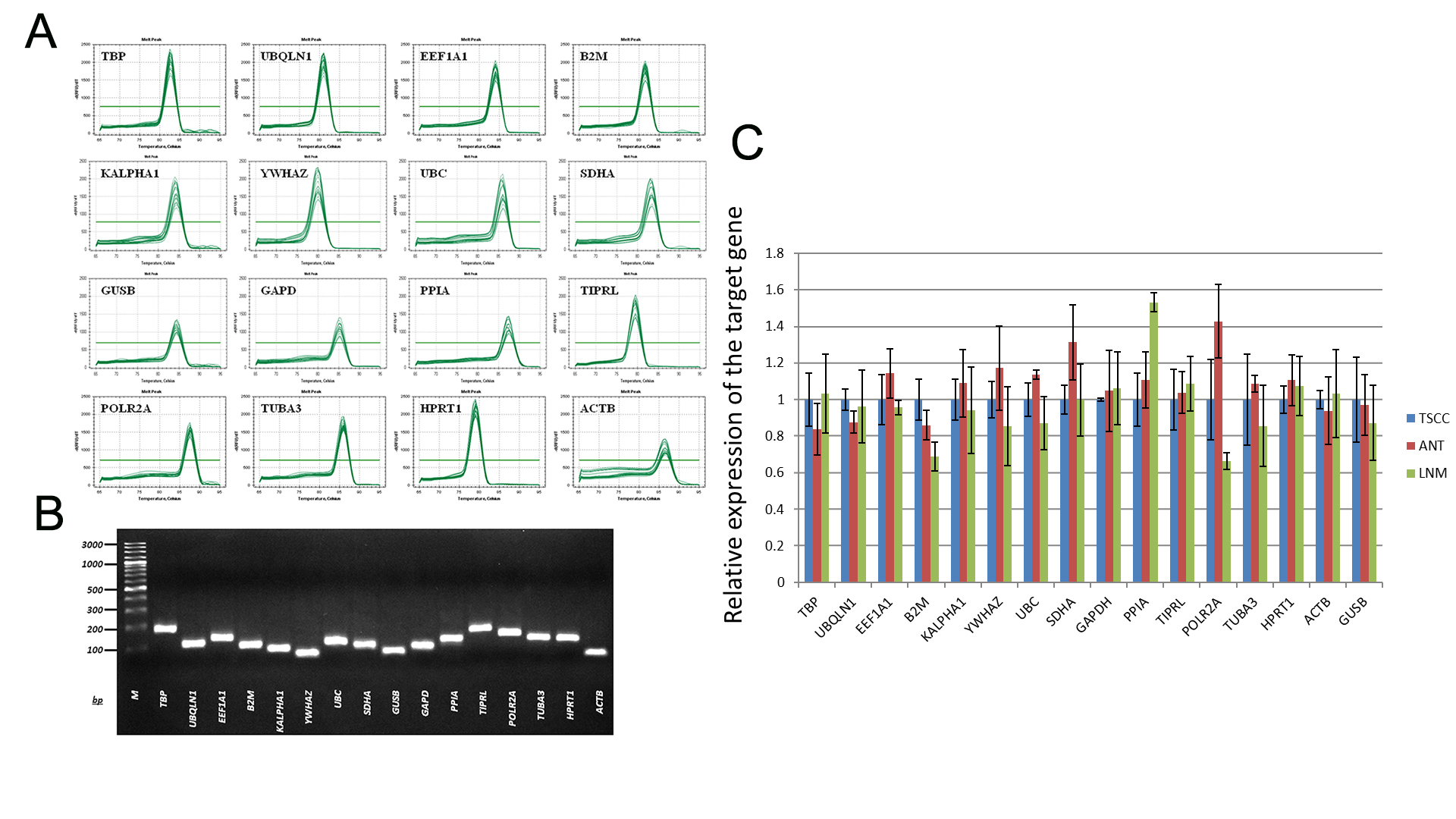

Supplement: Additional file 2: Figure S1. — References gene selection for the paired TSCC, ANT and LNMs. A: Melting curve of the amplification of the targeted genes; B: Gel electrophoresis of the amplified products in Figure S1A.; C: Column diagram with SD bar illustrated the relative expression of targeted genes as a ratio of ANT/LNM to paired primary tumor. (JPG 718 kb) [file 12885_2016_2735_MOESM2_ESM.jpg]

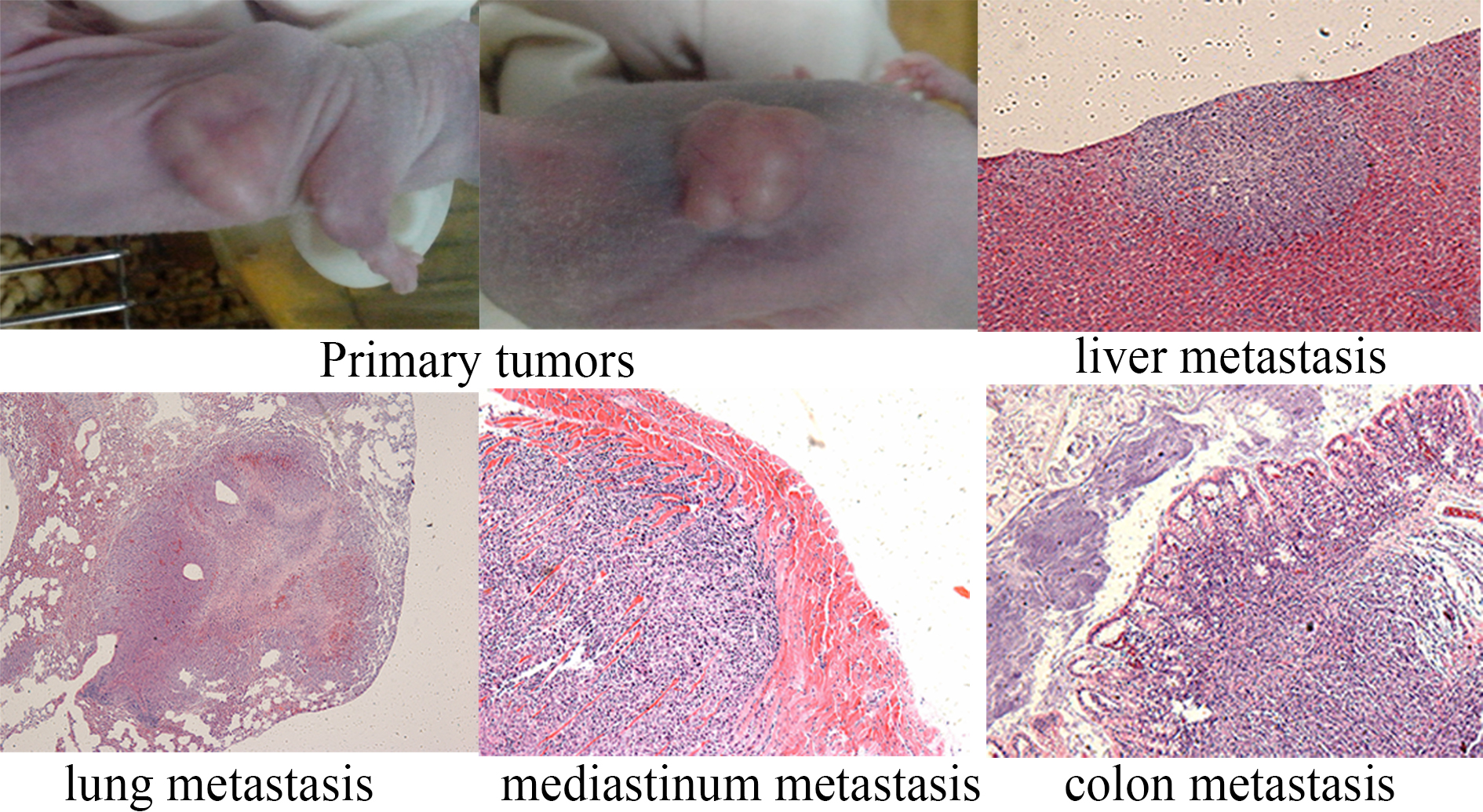

Supplement: Additional file 3: Figure S2. — Establishment of the SCC metastases animal model in nude mice; grossly obvious tumors and metastases were dissected and fixed immediately with 4 % paraformaldehyde for pathological analysis. (JPG 1505 kb) [file 12885_2016_2735_MOESM3_ESM.jpg]

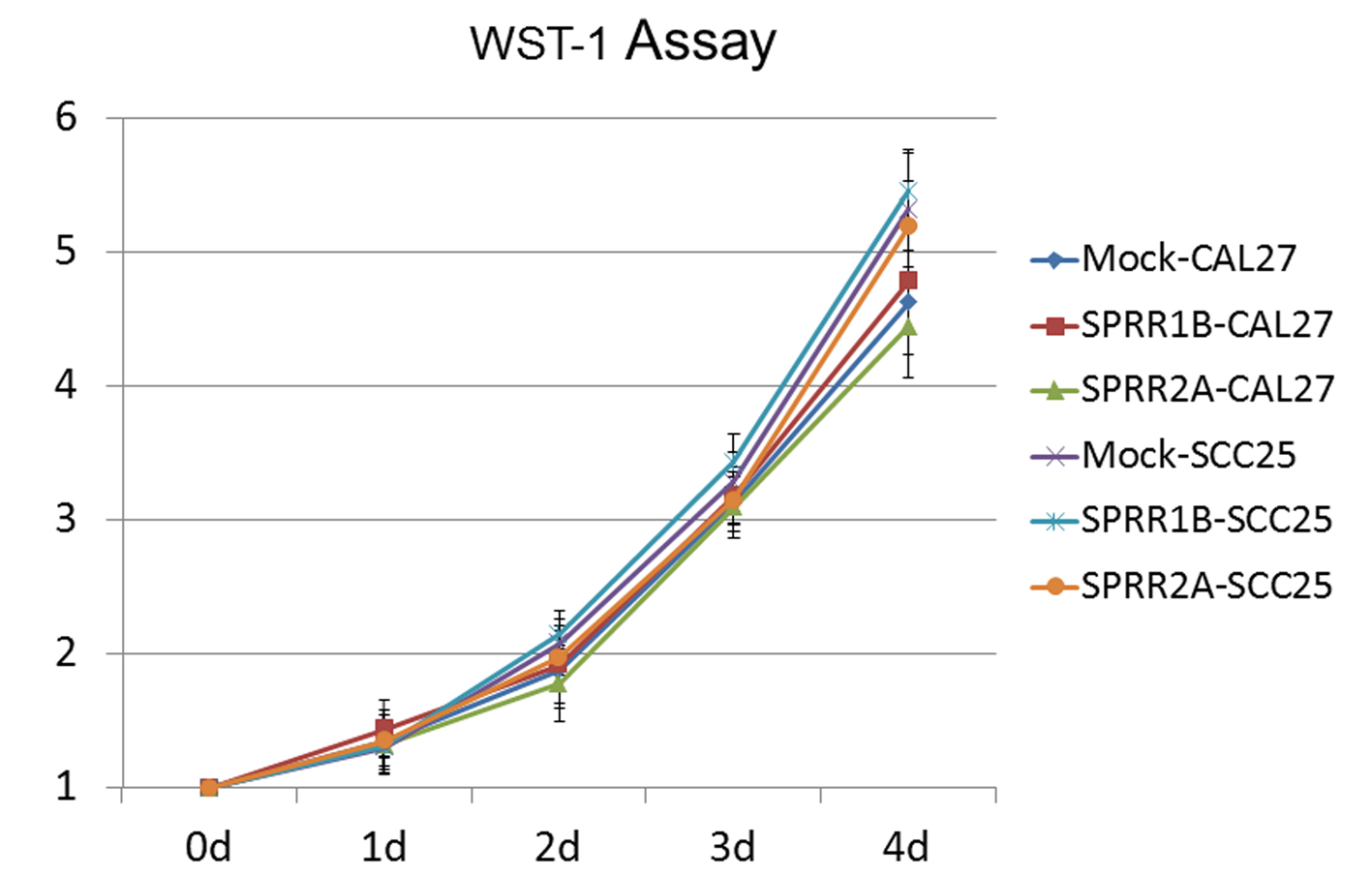

Supplement: Additional file 4: Figure S3. — WST-1 (Roche) assay measuring the activity of mitochondrial dehydrogenases was performed following the manufacturer’s instruction at 0-, 1-, 2-, 3-, 4- day time points. Error bars represent the standard deviation of the mean. (JPG 261 kb) [file 12885_2016_2735_MOESM4_ESM.jpg]

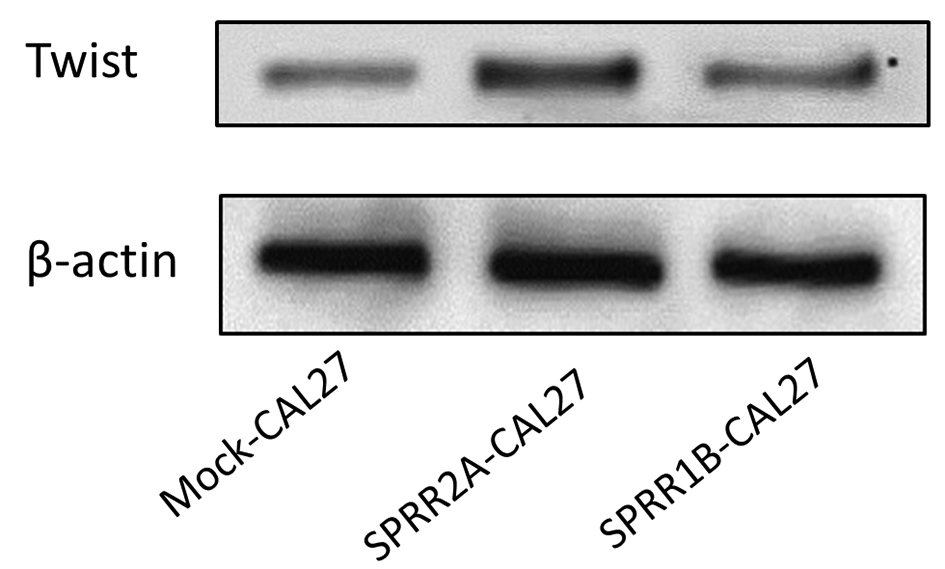

Supplement: Additional file 5: Figure S4. — Western blotting was performed to examine the protein levels of Twist in the indicated cells; β-actin was used as control. (JPG 151 kb) [file 12885_2016_2735_MOESM5_ESM.jpg]
